# BMAL1 regulates mitochondrial fission and mitophagy through mitochondrial protein BNIP3 and is critical in the development of dilated cardiomyopathy

**DOI:** 10.1007/s13238-020-00713-x

**Published:** 2020-04-10

**Authors:** Ermin Li, Xiuya Li, Jie Huang, Chen Xu, Qianqian Liang, Kehan Ren, Aobing Bai, Chao Lu, Ruizhe Qian, Ning Sun

**Affiliations:** 1 Department of Physiology and Pathophysiology, State Key Laboratory of Medical Neurobiology, School of Basic Medical Sciences, Fudan University, Shanghai, 200032 China; 2 Department of Pathology, School of Basic Medical Sciences, Fudan University, Shanghai, 200032 China; 3 Shanghai Key Lab of Birth Defect, Children’s Hospital of Fudan University, Shanghai, 201102 China; 4 Shanghai Key Laboratory of Clinical Geriatric Medicine, Fudan University, Shanghai, 200032 China; 5 Research Center on Aging and Medicine, Fudan University, Shanghai, 200032 China

**Keywords:** circadian gene *BMAL1*, human embryonic stem cells, cell differentiation, cardiomyocytes, dilated cardiomyopathy, mitochondria

## Abstract

Dysregulation of circadian rhythms associates with cardiovascular disorders. It is known that deletion of the core circadian gene *Bmal1* in mice causes dilated cardiomyopathy. However, the biological rhythm regulation system in mouse is very different from that of humans. Whether *BMAL1* plays a role in regulating human heart function remains unclear. Here we generated a *BMAL1* knockout human embryonic stem cell (hESC) model and further derived human *BMAL1* deficient cardiomyocytes. We show that *BMAL1* deficient hESC-derived cardiomyocytes exhibited typical phenotypes of dilated cardiomyopathy including attenuated contractility, calcium dysregulation, and disorganized myofilaments. In addition, mitochondrial fission and mitophagy were suppressed in *BMAL1* deficient hESC-cardiomyocytes, which resulted in significantly attenuated mitochondrial oxidative phosphorylation and compromised cardiomyocyte function. We also found that BMAL1 binds to the E-box element in the promoter region of *BNIP3* gene and specifically controls BNIP3 protein expression. *BMAL1* knockout directly reduced BNIP3 protein level, causing compromised mitophagy and mitochondria dysfunction and thereby leading to compromised cardiomyocyte function. Our data indicated that the core circadian gene *BMAL1* is critical for normal mitochondria activities and cardiac function. Circadian rhythm disruption may directly link to compromised heart function and dilated cardiomyopathy in humans.

**Electronic supplementary material:**

The online version of this article (10.1007/s13238-020-00713-x) contains supplementary material, which is available to authorized users.

## Introduction

The circadian clock, also known as a circadian oscillator, is ubiquitously present in every cell and critical to regulate internal physiological rhythms and behaviors throughout an organism’s life. The body’s circadian clock produces a robust 24-hour rhythmic activity as the Earth rotates (Kang et al., 2009). These intrinsic circadian rhythms are based on transcriptional-translational feedback loops (TTFLs) to maintain organ homeostasis (Dierickx et al., 2018). The core TTFLs are driven by four intrinsic clock genes: two activation factors CLOCK and BMAL1, and two inhibitors Period (PER1, 2, 3) and Cryptochrome (CRY1,2) (Huang et al., 2012; Partch et al., 2014). In mammals, the central circadian clock is located in the suprachiasmatic nucleus of the hypothalamus, while the peripheral circadian clocks are present in peripheral organs and tissues (Balsalobre et al., 1998), including the heart (Maemura et al., 2001) and vasculatures (McNamara et al., 2001; Nonaka et al., 2001).

Recent studies revealed that dysregulation of circadian rhythms associates with metabolic and cardiovascular disorders (Mottillo et al., 2010; Maury et al., 2014). Global *Bmal1*-deficient mice displayed age-associated dilated cardiomyopathy (DCM), exhibiting left ventricular dilatation and contractile dysfunction (Lefta et al., 2012). This suggests that dysregulation of circadian rhythms may cause gradual abnormality of heart function and finally lead to cardiac dilation and failure. However, whether *BMAL1* deficiency causes a similar cardiac phenotype in humans is unknown. And, the molecular mechanisms of *BMAL1* deficiency leading to the DCM phenotype is also unclear.

DCM is one of the main causes of heart failure (Maron et al., 2006). So far, more than 50 kinds of single-gene mutations have been found to lead to DCM(Vikhorev et al., 2017). Gene mutations involving sarcomere (Gerull et al., 2002), nuclear envelope (Fatkin et al., 1999; Parks et al., 2008), transcriptional pathways (Pyun et al., 2018), ion channel (Schmitt et al., 2003) and mitochondrial (Song et al., 2015) proteins are the most common genetic causes of DCM. However, the etiology of some idiopathic DCM remains unclear. In this study, by analyzing the published gene expression omnibus (GEO) data (GSE42955), we found that the *BMAL1* mRNA level in advanced DCM patients was much higher than that of the normal people. We disrupted *BMAL1* gene expression in human embryonic stem cells (hESCs) using genomic editing techniques and further derived human cardiomyocytes by cardiac-specific differentiation. We found hESC-derived cardiomyocytes with *BMAL1* knockout (KO) exhibited abnormalities such as disruption of sarcomeres, enlarged cell size, impaired contractility and calcium handling, as well as arrhythmia. Cardiomyocytes from both *BMAL1* KO hESCs and *Bmal1* KO mice showed an increased mitochondrial fusion and a decline in mitochondrial autophagy, resulted in attenuated mitochondrial oxidative phosphorylation and mitochondrial function. We further discovered that BMAL1 binds to the E-box element in the promoter region of the mitochondrial gene *BNIP3*. Knocking out *BMAL1* caused a decrease in BNIP3 protein expression, which partly induced impaired mitochondrial fission and mitophagy, leading to compromised cardiomyocyte function and the macroscopic phenotype of DCM.

Overall, our results showed that the circadian gene *BMAL1* plays a critical role in maintaining normal function of cardiomyocytes and the heart. Circadian rhythm disruption may directly link to compromised heart function and dilated cardiomyopathy in humans.

## Results

### *Bmal1* deficient mice developed dilated cardiomyopathy

**Figure 1:**
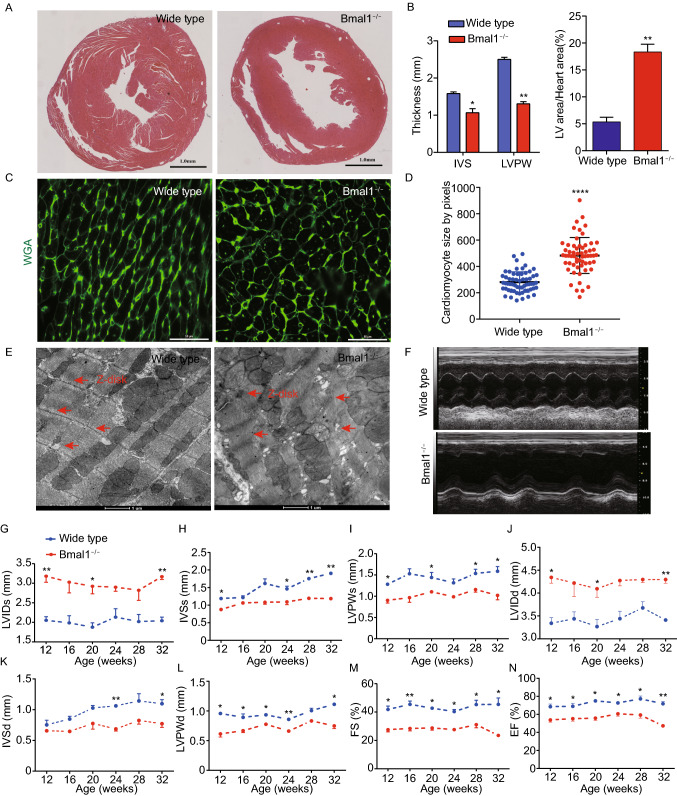
***Bmal1*** **deletion caused dilated cardiomyopathy in mice**. (A) Histological analyses of heart sections of wild type and *Bmal1* KO mice by H&E staining at 32 weeks of age. Scale bar: 1 mm. (B) Statistics of the average thickness of IVS and LVPW of wild type and *Bmal1* KO mice (*n* = 4). *P < 0.05 and **P < 0.01 versus control by two-tailed Student’s *t* test. (C) Representative confocal microscopy images of WGA staining of myocardium from wild type and *Bmal1* KO mice. (D) Quantification of cell surface area as shown in (C) (*n* = 59 per group). ****P < 0.0001 versus control by two-tailed Student’s *t* test. (E) Representative electron micrographs of ventricular cardiomyocytes from wide type and *Bmal1* KO mice. Red arrows indicate Z-lines. (F) Representative echocardiography images of 32-weeks-old wild type and *Bmal1* KO mice. (G–N) Echocardiographic parameters of heart functions from wild type and *Bmal1* KO mice over time (*n* = 4 per group). 2-way ANOVA with post-hoc tes. Data were represented mean ± SD. *P < 0.05 and **P < 0.01 versus control by 2-way ANOVA with post-hoc test

To see whether the circadian clock gene *Bmal1* plays a role in cardiac function, we generated mice with global deletion of the *Bmal1* gene (Fig. S1) and examined their heart morphology and function over time. Mice with *Bmal1* KO showed measurable weight loss and heart rate reduction during their growth (Fig. S2A and S2B). At 32 weeks of age, histological analyses of cross sections of the heart at the level of papillary muscles showed that *Bmal1* deficient mice displayed a larger left ventricular cavity plus thinning of left ventricular posterior wall and interventricular septum (Fig. 1A and 1B). The myocyte cross-sectional area was also increased in *Bmal1* KO mice when compared to that of the wild type mice (Fig. 1C and 1D), indicating dilation of the myocardium and the ventricle. In addition, transmission electron microscopy showed that *Bmal1* KO mice exhibited disorganization and compaction of sarcomeres. The sarcomeric Z-lines were also become condensed and wider (Fig. 1E).

From 12-weeks of age, we assessed heart functions of wild type and *Bmal1* KO mice by echocardiography at four-weeks intervals (Fig. 1F). *Bmal1* KO mice showed a significantly increased left ventricular end systolic internal diameter (LVIDs) at 32 weeks compared to that in wild type mice (Fig. 1G). The interventricular septum thickness at end-systole (IVSs) and left ventricular posterior wall thickness at end-systole (LVPWs) also gradually became thinner in *Bmal1* KO mice than those in wild type mice (Fig. 1H and 1I). Increased left ventricular internal dimension at end-diastole (LVIDd), decreased interventricular septum thickness at end-diastole (IVSd), and left ventricular posterior wall thickness at end-diastole (LVPWd) were also observed in *Bmal1* KO mice (Fig. 1J–L). Further, *Bmal1* KO mice showed impaired cardiac contractility with a decreased fraction of shortening (FS) (Fig. 1M) and ejection fraction (EF) (Fig. 1N). Overall, *Bmal1* KO led to a dramatic ventricular dilation and a ~50% reduction in cardiac contractility in mice at later age. These results are consistent with previous reports showing *Bmal1* deficiency induced age-associated DCM in mice (Lefta et al., 2012).

### *BMAL1* deficient hESC-derived cardiomyocytes exhibited myofilament sarcomere disruption and increased apoptosis

**Figure 2:**
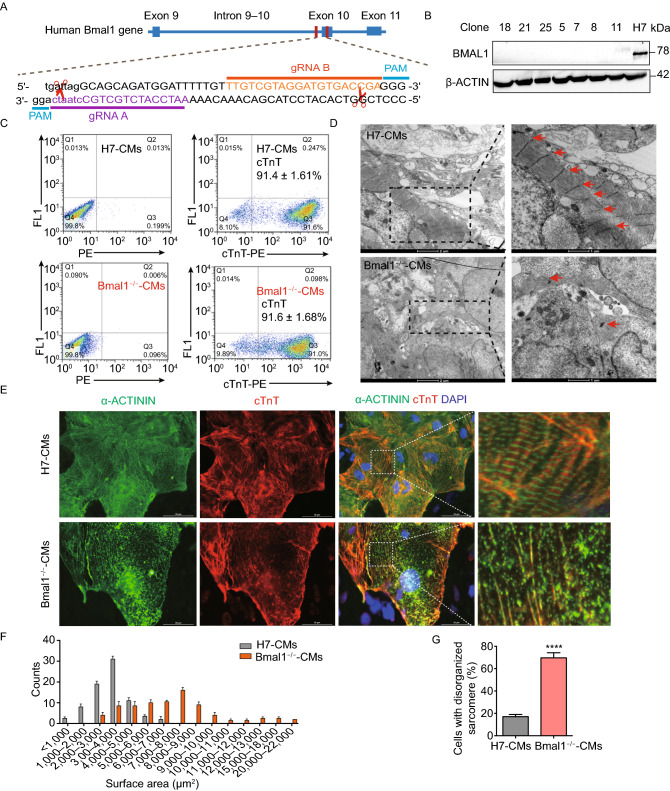

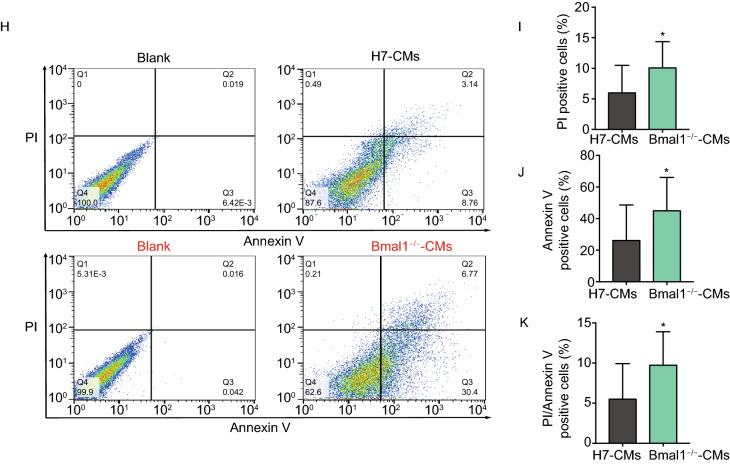
**Phenotypic characterizations of** ***BMAL1*** **KO hESCs-derived cardiomyocytes**. (A) Schematic showing the design for generation of *BMAL1* KO hESC cell line by genomic editing with CRISPR/Cas9 technique. The CRISPR/Cas9 cutting site is on exon 10 of human *BMAL1* gene. gRNA: guide RNA, PAM: protospacer adjacent motif. (B) Western blot of BMAL1 protein expression in wild type hESCs and *BMAL1* KO positive hESC clones. (C) Flowcytometry analyses of cardiac differentiation efficiency of wild type and *BMAL1* KO hESCs. Cardiomyocytes were stained with the classic cardiac marker cardiac troponin T (*cTnT*). (D) Representative transmission electron micrographs of sarcomeric structures in wild type and *BMAL1* KO hESC-derived cardiomyocytes. Scales bar: 1 µm. (E) Immunostaining of cTnT and α-Actinin in wild type and *BMAL1* KO hESC-derived cardiomyocytes. Scale bars: 50 µm. (F) Quantification of cell size for wild type and *BMAL1* KO hESC-derived cardiomyocytes (*n* = 85 per group). (G) Higher percentage of disorganized sarcomeres for *BMAL1* KO hESC-derived cardiomyocytes. (H) Detection of apoptosis by flowcytometry in wild type and *BMAL1* KO hESC-derived cardiomyocytes. (I–K) Quantification of the ratio of annexin V^−^/PI^+^ cells, annexin V^+^/PI^−^ cells and annexin V^+^/PI^+^ cells. Data were represented as means ± SD. *P < 0.05 and ****P < 0.0001 versus control by two-tailed Student’s *t* test

To further assess the role of *BMAL1* in human cardiomyocytes, we knocked out *BMAL1* expression in H7 hESCs by CRISPR/Cas9 mediated genome editing (Fig. 2A). Indel mutations were introduced within exon 10 of *BMAL1* by CRISPR/Cas9 nickase, which led to frameshifts and creation of a premature stop codon in *BMAL1* locus (Fig. S3A–C). Western blot results indicated that BMAL1 protein expression was knocked out in the selected positive clones (Fig. 2B). In order to confirm whether both alleles of the *BMAL1* gene were disrupted, we performed TA cloning of the amplified PCR products of the target cutting region and it showed that those positive clones were all homozygous. The two alleles of the clone 18 had the same genetic modification (Fig. S3D), while the others had two different genetic mutations (data not shown). The established *BMAL1* KO hESC lines exhibited normal ES-cell-like morphologies and pluripotency nature (Fig. S4). Although *BMAL1* KO hESCs spontaneously differentiated into the three germ layers in teratoma assays, an upregulation of the mesodermal, cardiac progenitor, and endodermal genes were observed (Fig. S5).

We further differentiated these *BMAL1* KO hESCs into beating cardiomyocytes (Supplementary Movie 1 and 2) (Lian et al., 2012). The differentiation efficiency regularly reached greater than 90% (Fig. 2C). To evaluate the impact of *BMAL1* KO on cardiomyocyte morphology and myofilament structure, immunofluorescence stainings were performed and it showed that *BMAL1* KO hESC-cardiomyocytes exhibited abnormal sarcomeric cardiac troponin T (cTnT) and α-Actinin distribution (Fig. 2E). Assessing ultra-structures using transmission electron microscopy further showed that wildtype hESC-cardiomyocytes exhibited well organized sarcomeres and regular Z-lines, while *BMAL1* KO hESC-cardiomyocytes displayed severe myofilament disorganization and irregular sarcomeric Z-lines (Fig. 2D). We also assessed the cell size of both widetype mice and *BMAL1* KO hESC-cardiomyocytes. The average cell area is significantly larger in *BMAL1* KO hESC-cardiomyocytes (wildtype hESC-cardiomyocytes, 3,370 ± 133.6 μm^2^; *BMAL1*^−/−^ hESC-cardiomyocytes, 6,965 ± 396.3 μm^2^, *n* = 85 per group, *P* < 0.001) (Fig. 2F). Compared with wild type hESC-cardiomyocytes, the percentage of *BMAL1* deficient hESC-cardiomyocytes with disorganized sarcomeres increased significantly (Fig. 2G). In addition, *BMAL1* deficiency led to a significant increase in both early apoptosis and late apoptosis according to flowcytometry analysis (Fig. 2H–K). These data indicated that *BMAL1* KO had a great impact on myofilament organization and morphology of human cardiomyocytes.

### Compromised contractility and calcium handling in *BMAL1* KO cardiomyocytes

**Figure 3:**
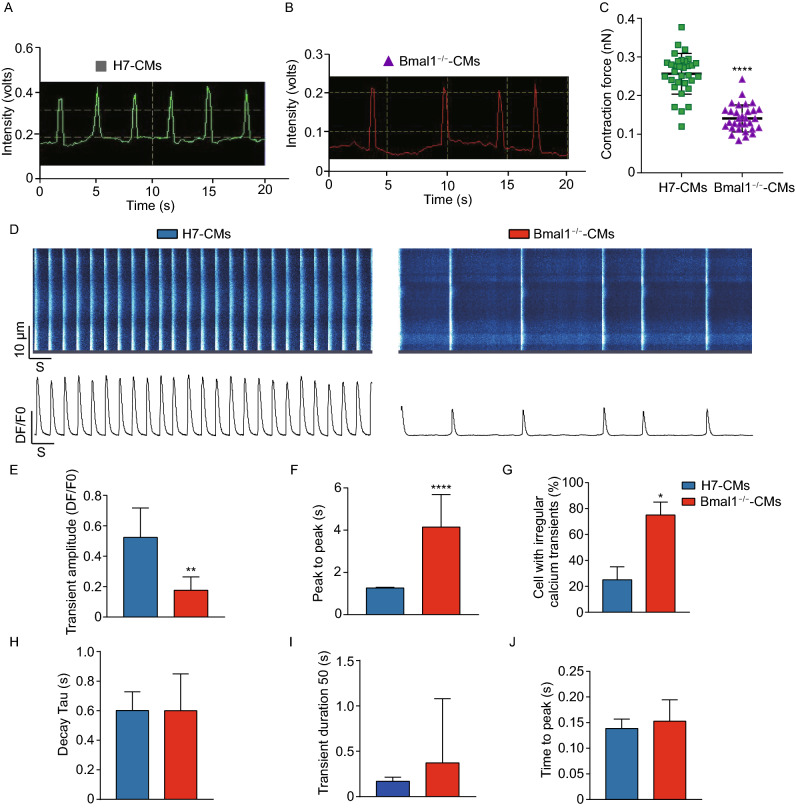
**Compromised contraction force and abnormal calcium handling in** ***BMAL1*** **KO hESC-derived cardiomyocytes**. (A) Representative traces of video detection for contraction movements from single wild type hESC-derived cardiomyocytes day 35 post differentiation. (B) Representative traces of video detection for contraction movements from single *BMAL1* KO hESC-derived cardiomyocytes day 35 post differentiation. (C) Quantification of relative contraction force of single wild type and *BMAL1* KO hESC-derived cardiomyocytes (*n* = 32 per group). (D) Representative Ca^2+^ line scan images and spontaneous Ca^2+^ transients in wild type and *BMAL1* KO hESC-derived cardiomyocytes day 35 post differentiation. (E–J) Quantification of calcium handling parameters in wild type and *BMAL1* KO hESC-derived cardiomyocytes. E, transient amplitude (average ΔF/F0). F, peak to peak time. G, ratio of cardiomyocytes with irregular Ca^2+^ transients. H, decay time. I, transient duration 50. J, time to peak. *n* = 25 in each group. Data were represented as mean ± SD. *P < 0.05, **P < 0.01 and ****P < 0.0001 versus control by two-tailed Student’s *t* test

Our results showed that *Bmal1* knockout mice exhibited reduced heart contractility and cardiomyocyte sarcomeric disruption. To assess whether *BMAL1* KO has an impact on contractility of human cardiomyocytes, we next measured the contraction force of single *BMAL1* KO hESC-derived cardiomyocytes. We found that single *BMAL1* KO hESC-cardiomyocytes exhibited weakened contractile force generation per cell movement and irregular beating rhythm when compared with wildtype control hESC-cardiomyocytes (Fig. 3A–C).

Previous studies on Ca^2+^ homeostasis showed that calcium handling plays an important role in excitation-contraction coupling and subsequent force generation of cardiomyocytes (Cheng et al., 1993; Bers, 2002; Eisner et al., 2017). Myocardium from failing hearts also showed defective excitation-contraction coupling which contributes to impaired contractility (Gomez et al., 1997; Yano et al., 2000; Piacentino et al., 2003). We then explored the Ca^2+^ handling property of *BMAL1* KO hESC-cardiomyocytes by calcium imaging. The results showed that *BMAL1* KO hESC-cardiomyocytes exhibited more irregular Ca^2+^ transients compared with those of wild type control hESC-cardiomyocytes (Fig. 3D and 3G). Further, *BMAL1* KO hESC-cardiomyocytes showed a reduction in Ca^2+^ transient amplitude, indicating that *BMAL1* deficiency weakened myofilament Ca^2+^ sensitivity (Fig. 3E). *BMAL1* KO hESC-cardiomyocytes also showed a slower peak to peak time, indicating a significantly elongated beating activity (Fig. 3F). There was no significant difference in the relative time to peak and Ca^2+^ decay kinetics between wildtype and *BMAL1* KO hESC-cardiomyocytes (Fig. 3H–J). These data indicated that *BMAL1* KO impaired contractility and calcium handling of hESC-cardiomyocytes.

### Electrophysiological properties of *BMAL1* KO hESC-cardiomyocytes

**Figure 4:**
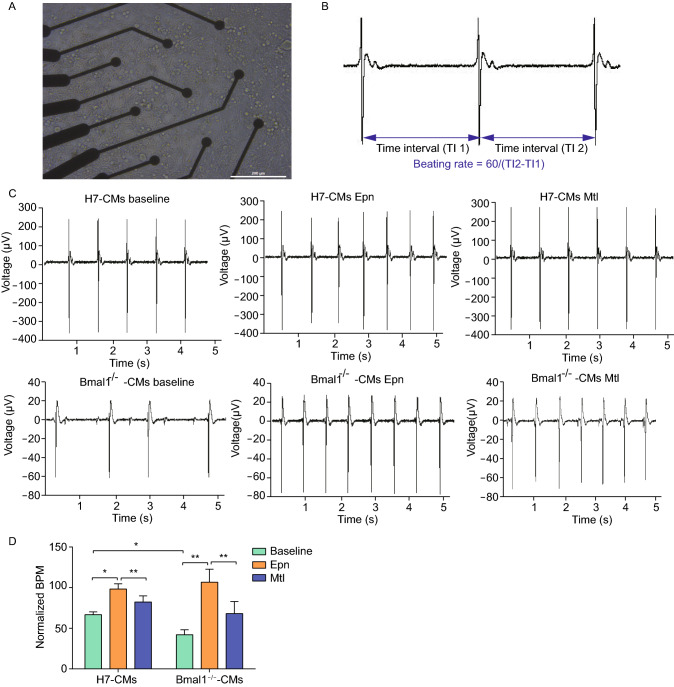
**Multi-electrode array analyses of electrophysiology of** ***BMAL1*** **KO hESC-derived cardiomyocytes**. (A) hESC-derived cardiomyocytes day 35 post differentiation cultured on a MEA probe coated with matrigel. Scale bar: 200 μm. (B) Typical field potential traces of hESC-derived cardiomyocytes recorded by MEA. (C) Representative MEA recordings showed field potential traces from wild type and *BMAL1* KO hESC-derived cardiomyocytes day 35 post differentiation. (D) Quantification of beating frequency of wild type and *BMAL1* KO hESC-derived cardiomyocytes in response to epinephrine and metoprolol. Data were represented as mean ± SD. The statistics were from 3 independent experiments. *P < 0.05 and **P < 0.01 versus control by two-tailed Student’s *t* test

Previous studies revealed that sympathetic nervous system plays a key role in regulating cardiac physiology(Hoover et al., 2004) by modulating the heart rate, contractility (Li et al., 2000; Shan et al., 2010), and structure of myocytes (Kanevskij et al., 2002; O’Connell et al., 2003; Kreipke and Birren, 2015). To evaluate the effect of *BMAL1* deficiency on cardiomyocytes to neuro-hormonal regulation, non-invasive microelectrode arrays (MEAs) was utilized to analyze pharmacologic effects of heart-related drugs on hESC-cardiomyocytes. The drugs that can influence sympathetic nervous system, for instance adrenergic catecholamine norepinephrine (NE) and beta-adrenergic receptor agonist metoprolol (Mtl), were used to measure cardiac electrophysiological characteristics. Wildtype or *BMAL1* KO hESC-cardiomyocytes day 30 post differentiation were seeded on MEA probe and the monolayer cardiomyocytes on the MEA surface began to beat spontaneously after 24 h in culture. Synchronous and stable traces for electrophysiological parameters were recorded on day 35 (Fig. 4A). Parameters of MEAs used for the evaluation were characterized by beating rate (Fig. 4B). Irregular beating activities were observed in the onset of *BMAL1* KO hESC-cardiomyocytes (Fig. 4C). Moreover, baseline electrophysiological parameters showed that the average beating frequency of *BMAL1* KO hESC-cardiomyocytes was much lower than that of the wild type hESC-cardiomyocytes (Fig. 4D).

### Mitochondrial fission and autophagy were compromised in *BMAL1* KO hESC-cardiomyocytes and in the myocardium of *Bmal1*-deficient mice

**Figure 5:**
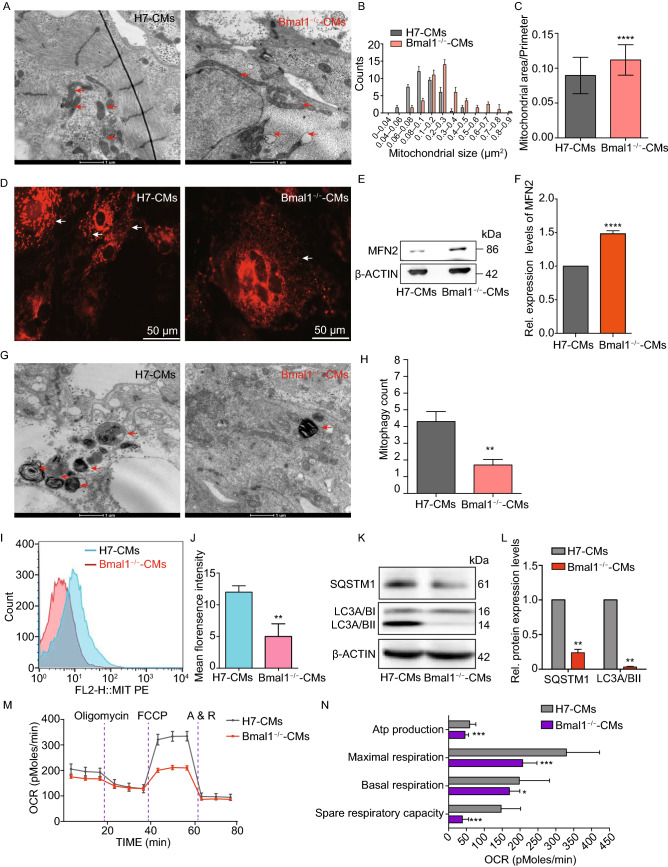
***BMAL1*** **KO hESC-derived cardiomyocytes exhibited mitochondrial hyperfusion and compromised mitochondrial autophagy**. (A) Representative transmission electron micrographs of mitochondria (red arrows) from wild type and *BMAL1* KO hESC-derived cardiomyocytes day 35 post differentiation. Mitochondrial enlargement in *BMAL1* KO hESC-derived cardiomyocytes was apparent. (B) Quantification of mitochondrial size in wild type and *BMAL1* KO hESC-derived cardiomyocytes day 35 post differentiation. (C) Quantification of the ratio of mitochondrial area-perimeter indicated mitochondrial fusion level (enlarged and fused mitochondria possess greater area-perimeter ratio) was upregulated in *BMAL1* KO hESC-derived cardiomyocytes (*n* = 40). (D) Representative confocal images showing RFP-tagged mitochondria (white arrows) in wild type and *BMAL1* KO hESC-derived cardiomyocytes day 35 post differentiation. Cox8a-RFP adenovirus was used to tag mitochondria. (E) Western blot analysis of level of Mfn2 proteins regulating mitochondrial dynamics. (F) Quantification of Western blot signals in (E) and normalized to the loading control (α-ACTIN). (G) Transmission electron microscopy examining alterations in mitochondrial autophagy (red arrows) in wild type and *BMAL1* KO hESC-derived cardiomyocytes day 35 post differentiation. (H) Mitophagy was counted in 10 different transmission electron images of wild type and *BMAL1* KO hESC-derived cardiomyocytes day 35, respectively. (I) Mitophagy intensity in wild type and *BMAL1* KO hESC-derived cardiomyocytes day 35 post differentiation analyzed by flowcytometry. (J) Mean intensity of *BMAL1* KO hESC-derived cardiomyocytes was markedly decreased. (K) Western blot evaluation of protein expression level of SQSTM1 and LC3A/B-II in wild type and *BMAL1* KO hESC-derived cardiomyocytes day 35 post differentiation. (L) Quantification of Western blot signals in (I) and normalized to the loading control (α-ACTIN). *n* = 3 for each group. (M) Real-time respiration measurements of wild type and *BMAL1* KO hESC-derived cardiomyocytes day 35 post differentiation. (N) Statistics of respiration parameters between wild type and *BMAL1* KO hESC-derived cardiomyocytes day 35 post differentiation. *n* = 4 for each group. Data were represented as mean ± SD. *P < 0.05, **P < 0.01, ***P < 0.001, ****P < 0.0001 versus control by two-tailed Student’s *t* test

**Figure 6:**
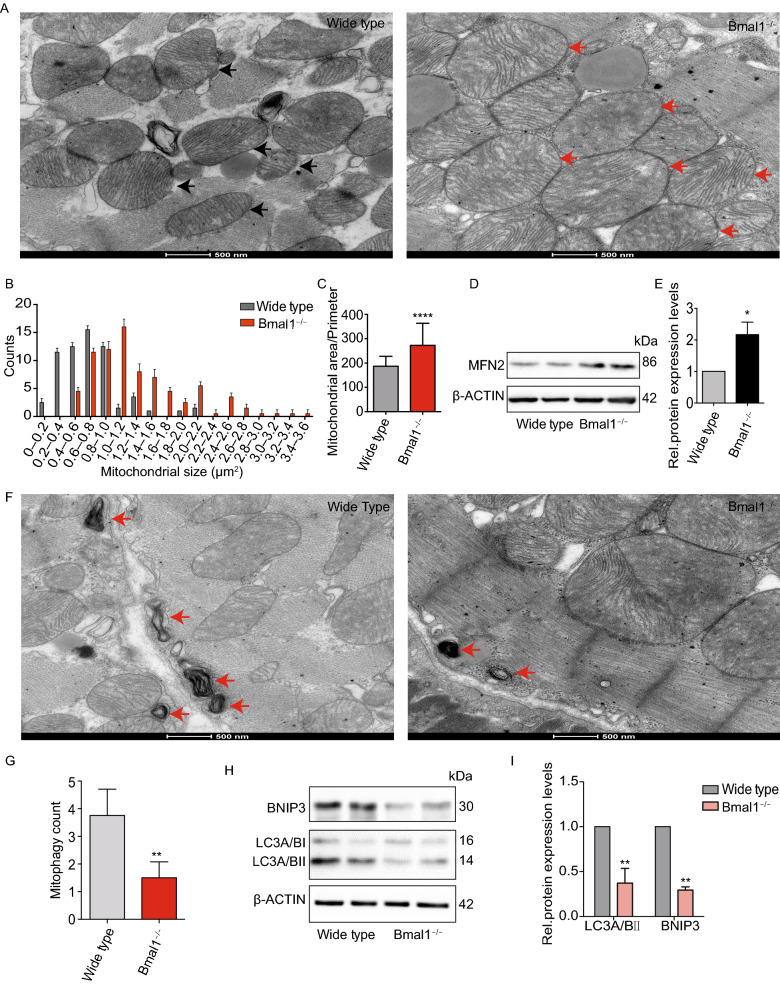
M**itochondria in cardiomyocytes of** ***Bmal1*-deficient mice exhibited increased fusion and decreased mitophagy**. (A) Significantly enlarged mitochondria were observed in *Bmal1*-deficient myocardium comparing to those in the wild type group. Arrows indicate mitochondria. (B and C) Mitochondrial size and area-perimeter ratio were carried out for the quantification of mitochondrial fusion level (enlarged and fused mitochondria possess greater area-perimeter ratio). (D) The protein expression level of MFN2 was calculated by western Blotting analysis evaluation (expressed as the ratio of β-ACTIN). (E) Columns in graphs show protein normalized for β-ACTIN. (F) Less mitophagy take place in Bmal1^−/−^ mice myocardium than in wild type control group. (G) Mitophagy were counted in 12 different transmission electron images of *Bmal1*^−/−^ mice myocardium and wild type control group, respectively. (H and I) The protein expression level of LC3A/BI/IIand BNIP3 were assessed by Western Blotting analysis evaluation (expressed as the ratio of LC3A/BII and BNIP3 to β-ACTIN). Data represented the mean ± SD. *P < 0.05, **P < 0.01 and ****P < 0.0001 versus wide type by two-tailed Student’s *t* test

Mitochondrial dysfunction is considered to be a critical mechanism underlying cardiovascular diseases (Wallace, 2013; Murphy et al., 2016; Friederich et al., 2018). Abnormal mitochondrial morphology was observed in some mitochondrial diseases and idiopathic cardiomyopathies (McManus et al., 2019) . To see whether *BMAL1* ablation causes abnormality in mitochondrial function, we analyzed the ultrastructure of day 35 *BMAL1* KO hESC-cardiomyocytes by transmission electron microscopy. Mitochondria of *BMAL1* KO hESC-cardiomyocytes were extensively elongated, with abnormal cristae structures and an increased mitochondrial area/perimeter ratio (Fig. 5A–C). The most common intra-mitochondrial cristae malformations were circle cristae, partitioning cristae, swollen cristae, and cristolysis (Fig. S6). These ultrastructural mitochondria morphologies were consistent with previous findings (Jacobi et al., 2015; McManus et al., 2019). Immunostaining of *BMAL1* KO hESC-cardiomyocytes with adCox8a-RFP showed that mitochondria were highly hyper-fusion compared to those of wildtype hESC-cardiomyocytes (Fig. 5D, and Supplementary Movie 3 and Movie 4). Likewise, expression level of MFN2, the marker of mitochondrial fusion, was significantly increased in *BMAL1* KO hESC-cardiomyocytes (Fig. 5E and 5F). Similar findings were also present in *BMAL1* KO mice (Fig. 6A–E). Taken together, these results indicated that *Bmal1* deficiency led to hyper-fused mitochondria with abnormal cristae in cardiomyocytes.

A previous study demonstrated that the heart is one of the sturdiest mitophagic organs (Kanevskij et al., 2002). Mitophagy plays a crucial role in mitochondrial quality control, by removing dysfunctional, damaged, or aging mitochondria (Song et al., 2015; Song et al., 2017). Transmission electron microscopy imaging showed decreased mitophagy events in *BMAL1* KO hESC-cardiomyocytes (Fig. 5G and 5H). We thus further examined mitophagy intensity in wildtype and *BMAL1* KO hESC-cardiomyocytes using Mtphagy Dye (Iwashita et al., 2017). Consistent with transmission electron microscopy imaging, knocking out *BMAL1* resulted in a decline in mitophagy intensity (Fig. 5I and 5J). Mitochondrial localization of the autophagy chaperone protein SQSTM1 can mediate autophagy of damaged mitochondria by binding to autophagosomal microtubule associated protein1 light chain 3 (LC3) (Narendra et al., 2010). We found that mitochondrial SQSTM1 and LC3 II protein level were also decreased in *BMAL1* KO hESC-cardiomyocytes (Fig. 5K and 5L). Similar results were found in *Bmal1* KO mice (Fig. 6F–I,). In addition, mitochondrial autophagy-associated protein BNIP3 expression was reduced in myocardium of *Bmal1*-deficient mice (Fig. 6H–L). These data indicated that *BMAL1* ablation caused mitochondria abnormalities in and reduced mitophagy of cardiomyocytes. We further found that mitochondrial oxidative metabolisms of *BMAL1* KO hESC-cardiomyocytes were significantly declined (Fig. 5M and 5N). Overall, our data showed that *BMAL1* loss-of-function induced dysfunction and diminished respiration in mitochondria, suggesting that *BMAL1* deletion impacted the dynamic remodeling of the mitochondrial network.

### BMAL1 regulates mitophagy in cardiomyocytes partially by binding to the E-box elements in the promoter of *BNIP3* gene

**Figure 7:**
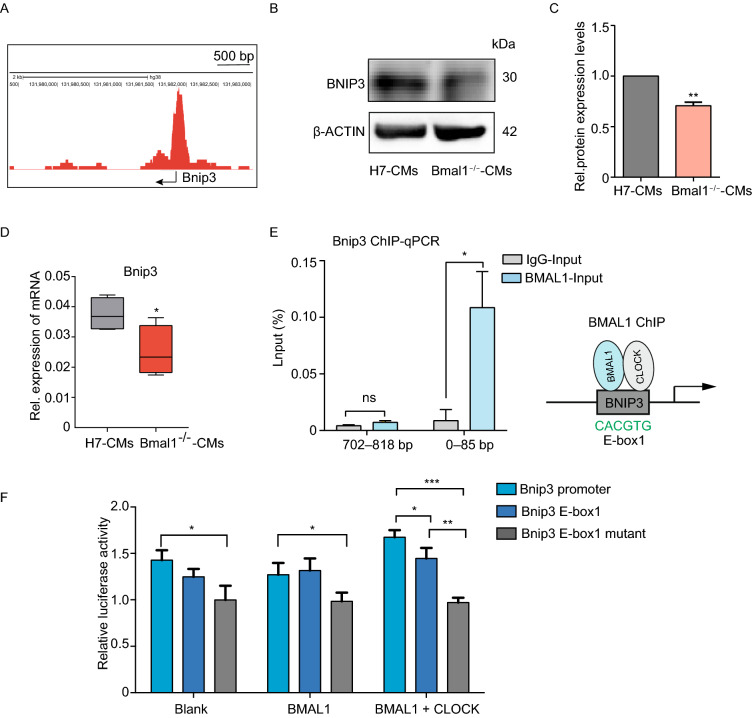
***BNIP3*** **is controlled by circadian gene** ***BMAL1*** **via transcription regulation**. (A) BMAL1 ChIP-seq showed binding signals on the promoter region of *Bnip3* in human U2OS cells (CistromeDB: 71023). The arrow indicates transcription start site (TSS) of the *Bnip3* gene. (B) Relative expression of *Bnip3* was analyzed in wild type and *BMAL1* KO hESC-derived cardiomyocytes day 35 post differentiation. (C) Western Blot showed that the expression of BNIP3 in *BMAL1* KO hESC-derived cardiomyocytes was significantly lower than that in wild type cardiomyocytes. (D) The mRNA levels of *BNIP3* in *BMAL1* KO hESC-derived cardiomyocytes day 35 post differentiation decreased significantly. (E) ChIP analysis of *Bmal1* binding to the E-box region of *BNIP3* in hESC-derived cardiomyocytes at day 35 post differentiation. (F) Dual luciferase reporter assays revealed that transcriptional activation of *Bnip3* in HEK 293 cells by BMAL1. Graph represented firefly luciferase expression normalized to renilla luciferase for each group. Data were mean ± SD from three biological replicates. *P < 0.05, **P < 0.01 and ***P < 0.001 versus control by two-tailed Student’s *t* test

**Figure 8:**
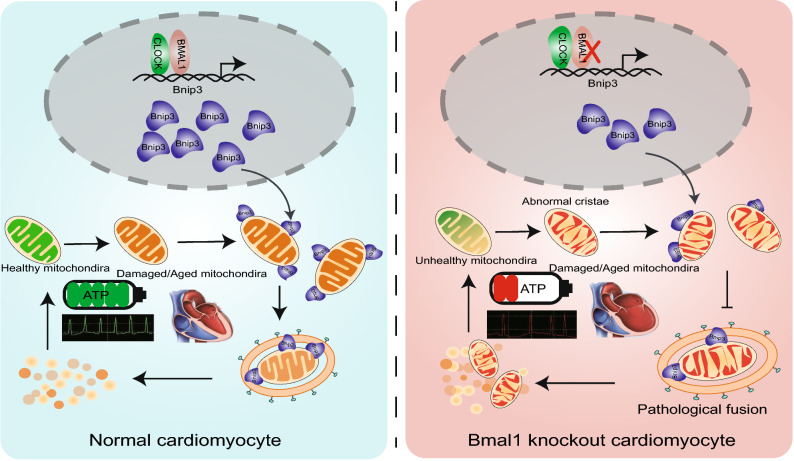
**The regulatory mechanism of** ***BMAL1*** **in mitophagy of cardiomyocyte and DCM**. Our study demonstrated that BMAL1 is able to bind to the E-box element in the promoter region of *BNIP3* gene and specifically control BNIP3 protein expression, hence directly affecting the *BNIP3*-mediated mitochondria quality control process. The circadian gene *Bmal1* is critical in the maintenance of normal mitochondria activities and cardiac function

According to genome-wide transcript profiling, BMAL1 is estimated to target more than 150 sites in the human genome, including all known clock genes and genes regulating metabolism (Hatanaka et al., 2010) . BMAL1 is a core circadian transcription factor and regulates downstream genes by binding to the E-box elements in their promoters (Ueda et al., 2005). The transcription of *Bnip3* gene exhibits a diurnal rhythm in C57 BL/6J livers (Jacobi et al., 2015). We also identified several canonical E-box sequences (CANNTG), and one noncanonical E-box sequence (CAGCTT) in the promoter region of *Bnip3* gene (Table. 1). In addition, BMAL1 is able to bind to the promoter region of *Bnip3* in osteosarcoma according to ChIP-seq analysis (Rey et al., 2016) (Fig. 7A). Since BNIP3 is a critical player in mitophagy by mediating autophagy of mitochondria and is located in myocardium (Azad et al., 2008; Chaanine et al., 2013; Xiao et al., 2018), we further examined BNIP3 protein expression in wild type and *BMAL1* KO hESC-cardiomyocytes at day 35. Western blot confirmed that BNIP3 protein level was reduced in *BMAL1* KO hESC-cardiomyocytes (Fig. 7B and 7C). The mRNA expression of *BNIP3* in *BMAL1* KO day 35 hESC-cardiomyocytes was also decreased (Fig. 7D). We therefore hypothesized that BMAL1 is responsible for the transcriptional activation of *BNIP3* by binding to the E-box elements within its promoter region in cardiomyocytes. ChIP-qPCR assays showed that there was a BMAL1 binding-site from 0 to 85 bp in the promoter region of *BNIP3* gene (Fig. 7E). We further used dual-luciferase reporter assays to evaluate the transcriptional activation of BMAL1 on *BNIP3*. As shown in Fig. 7F, wild type *BNIP3* promoter facilitated highest transcriptional activity to luciferase gene along with the co-transfection of *BMAL1* and *CLOCK*. However, E-box mutant *BNIP3* promoter was not able to sufficiently trigger the transcription of reporter gene with the exogenous overexpression of BMAL1 and CLOCK. These results indicated that BMAL1 regulates the transcription of *BNIP3* gene via binding with the E-Box element within its promoter region (Fig. 8).

## Discussion

The circadian oscillation, which is an endogenous time-keeping system, plays a key role in a wide range of physical processes by generation of 24-hour circadian rhythms in gene expression. Circadian clock mal-adjustment contributes to the pathology of various disorders, including insomnia (Flynn-Evans et al., 2017), aging (Tevy et al., 2013), cancer (Stevens, 2009), metabolic syndromes (Bray and Young, 2008), immune system imbalance (Deng et al., 2018), and cardiovascular diseases (Nonaka et al., 2001; Young et al., 2014). According to statistical studies, people with long-term irregular life routine have higher risk of hypertension, inflammation, and heart injury, which becomes more common in contemporary society (Morris et al., 2016). A number of studies reported that mutations in core circadian gene *BMAL1* or its partner *CLOCK* tended to develop cardiovascular diseases. One of the studies suggested *Clock*^*Δ19*^ mice displayed cardiac hypertrophy, impaired contractility, and reduced myogenic responsiveness (Alibhai et al., 2017). And, *Clock* specific mutant in cardiomyocytes resulted in myocardial contractile dysfunction and bradycardia (Bray et al., 2008). Different with the phenotypes of *Clock*^*Δ19*^ mice, loss of *Bmal1* gave rise to age-associated dilated cardiomyopathy, as a result of altered titin isoform component, change of *MYH* gene expression, and disrupted sarcomere structure (Lefta et al., 2012). Moreover, cardiomyocyte-specific *Bmal1* KO mice also gradually developed into dilated heart disease with age, partly through depressed glucose utilization as well as other cytologic mechanism related to metabolic malfunction (Young et al., 2014). It should be noted that these are all results from global *BMAL1* KO mice. Further study of cardiac-specific *BMAL1* KO animals are valuable for clarifying the precise role of *BMAL1* in the heart. However, rodents are nocturnal creatures, being active during the nights and sleeping during the day, which means their biorhythmic systems are opposite from human beings. And species difference between humans and mice may also give rise to different mechanisms in circadian system and cardiovascular diseases. It was actually not clear previously how *BMAL1* function in human cardiac system.

In this study, we successfully established *BMAL1* KO hESC cell lines and derived human *BMAL1*-deficient cardiomyocytes to mimic heart disorders associated with circadian malfunction (Young et al., 2001; Takeda and Maemura, 2010; Alibhai et al., 2017; Alibhai et al., 2018). In accordance with phenotypes in *Bmal1* KO mice, which displayed age-dependent dilated cardiomyopathy, *BMAL1*-deficient human cardiomyocytes showed characteristics of DCM including enlarged cell area, disrupted myofilament structure, increased apoptosis, attenuated contraction force, and dysfunction of calcium handling. We also found altered electrical conduction in our *BMAL1* KO cardiomyocytes as reported before, including deceased beating rates and irregular contractile rhythms. This suggested that, in addition to working cells, conduction-like cells may also be affected by *Bmal1* ablation.

In addition to disarrangement of sarcomere structure, morphological abnormalities of mitochondria in *BMAL1* KO cardiomyocytes were also observed by transmission electron microscopy, including mitochondrial swelling, sparse cristae, vacuolization, and abnormal elongation. Mitochondria function was also significantly impaired in *BMAL1* KO cardiomyocytes. Mitochondria events, including mitochondrial biogenesis, mitophagy, fusion and fission, are inseparably interconnected and play a significant role in the maintenance of normal mitochondria function. Mitochondrial biogenesis increases mitochondrial content levels to enhance the ability of energy metabolism. Fission process mediated by *Drp1* and fusion process mediated by *Mfn1*, *Mfn2*, and *Opa1* allow constantly remodeling of mitochondrial networks. And, mitophagy mediated by *Bnip3/NIX* in mammalian cells is a selective degradation process responsible for removal of damaged or aging mitochondria (Lee and Yoon, 2016; Vega and Kelly, 2017). Previous studies have shown that the dynamic balances in mitochondria were strictly regulated by biological rhythms. For example, mitochondrial fission-fusion turnover and metabolic flux is controlled by circadian activity of *Drp1* (Schmitt et al., 2018). In mice, circadian oscillation of NAD+ cycle prompted mitochondrial oxidative metabolism (Peek et al., 2013). Disturbed expression of circadian genes within cardiomyocytes leaded to structural and functional impairment of mitochondria(Bray et al., 2008). Our data is in accordance with these previous findings and suggested that *BMAL1* is critical for maintenance of normal mitochondrial structure and function.

Mitophagy is required not only to clear dysfunctional mitochondria, but also to adjust mitochondrial numbers for cellular energy needs. Disruption of autophagy or mitophagy has proved to be related to several cardiovascular disorders, such as myocardial infarction, cardiomyopathies, atherosclerosis, and cardiotoxicity (Bravo-San Pedro et al., 2017). In mammals, mitophagy process is mediated by NIX and its regulator BNIP3. Like many other BCL-2 proteins, BNIP3 participates in modulating the permeability of the outer mitochondrial membrane by forming homo- or hetero-oligomers, thus contributing to mitophagy. We found that *BMAL1* KO led to a reduced BNIP3 protein level and less mitophagosomes in hESC-derived cardiomyocytes. We also confirmed BMAL1 binds to one of the E-box elements in the promoter of *Bnip3* gene. Furthermore, *Bnip3* transcription was directly regulated by BMAL1. *Bmal1* deletion downregulated BNIP3 protein level, which compromised *Bnip3*-mediated mitophagy. Our data suggested that *Bmal1* ablation reduced BNIP3 expression and impaired normal mitochondrial quality control process, which might gradually induced abnormal cardiomyocyte function and phenotypes of dilated cardiomyopathy (Fig. 8). However, there are many idiopathic DCM cases in the clinic and the causes and detailed mechanism of this intractable disease remain unclear (Takahashi, 2017). Whether dysfunction of the circadian rhythms links to certain idiopathic DCM remains an interesting topic to clarify. Our results in this study provided a direct link between core circadian system and the disease of DCM.

Overall, our results showed that the circadian gene *BMAL1* plays a critical role in maintaining normal function of cardiomyocytes and the heart. Dysfunctional BMAL1 mutant protein may eventually lead to DCM in patients. It worth to check for *BMAL1* gene mutations using the whole genome sequencing technique and this could contribute to a clearer etiology diagnosis for clinical idiopathic DCM patients in the future.

## Materials and methods

### Human embryonic stem cells culture and differentiation

The human embryonic stem cell line H7 was cultured in mTeSR (STEMCELL TECHNOLOGIES, #85851 and #85852). HESC-H7 cells were isolated from matrigel by accutase digestion and reseeded on the new matrigel at 2–6 × 10^5^ cells per 12 well in mTeSR. We utilized the protocols originally developed by Lian et al (Lian et al., 2012) to achieve high-purity cardiomyocytes in vitro. Briefly, on the day of cardiac induction (day 0), 6 μmol/L CHIR99021 (http://www.selleckchem.com) was added for 2 days in 1 mL of insulin-minus RPMI + B27 medium. At day 2, cells were recovered in insulin-minus RPMI + B27 for 24 h. At day 3, cells were treated with 5 μmol/L IWR-1 (Sigma) for 2 days to activate Wnt signaling. At day 5, cells were finally switched to RPMI + B27 plus insulin medium. Beating cells were observed at day 7–8 after differentiation. The mature CMs were cultured in in DMEM containing 10% fetal bovine-serum. All these cells needed to be cultured on Matrigel-coated plates (corning #354248) and maintained at 37 °C with 5% CO_2_.

### CRISPR dual nickase editing

*BMAL1* gene mutation was corrected back to human embryonic stem cell line 7 (hESC-H7), using low off-target CRISPR dual nickase plasmid (PX462). Guide-RNA sequences (gRNA-A: 5′:AATCCATCTGCTGCCTAATCAGG; gRNA-B: 5′:TTGTCGTAGGATGTGACCGAGGG) were designed at http://crispr.mit.edu/ and cloned as described at http://www.genome-engineering.org/crispr/. The editing efficiencies of CRISPR plasmids were validated with 293T cells using Sanger sequencing. 4.6 × 10^5^ cells of hESC-H7 were electroporated using Neon Transfection System(Thermo Fisher Scientific) with 3 μg of the most efficient plasmids. Successful electroporation cells were puromycin (0.22 μg/mL) selected after 1 day. Single selected positive clones were picked and verified with Sanger sequencing using TA cloning technology to measure allelic frequency. We derived *BMAL1* gene mutation cell line during the CRISPR-editing process.

### EB formation

Human EB were generated as described previously(Ang et al., 2016). In ultra-low attachment plates (Corning), embryoid bodies can be formed in specific medium (DMEM, 20% FBS, 1% non-essential amino acid, 100 μmol/L BME, 1mmol/L L-glutamine) for 10 days followed by quantitative real-time PCR for evaluating gene expression of endoderm, mesoderm, ectoderm, cardiac progenitor, and mature cardiomyocytes.

### Animals

All animal procedures conformed to the National Institutes of Health Guidelines for the Use of Laboratory Animals were approved by Fudan University’s Animal Care and Use Committee guidelines. The germLine *Bmal1*^−/−^ mice were imported from Jackson Lab (Bunger et al., 2000). Mice were kept on a 14-h:10-h light-dark schedule and had access to food and water. Mice were anaesthetized using 1%–2% isoflurane and euthanized by cervical dislocation.

### Flow cytometry

The beating cardiomyocytes were digested with 1 mg/mL collagenase I (Sigma) from petri dishes, and followed by 0.25% Trypsin/EDTA (Gibco) to further isolate the cells. Then the isolated cells were fixed and infiltrated by permeabilization solution (BD Biosciences). The cells were stained for cardiac troponin T (cTnT, Thermo Scientific) for 2 h at 4 °C. The cells were incubated with the secondary antibody for 1 h before washing cells with PBS. Finally, the fluorescence-activated cell detection was performed on the FACSCalibur (BD Biosciences). The antibody information could be found in Table 2. Data was analyzed using FlowJo software.

### Mitophagy analysis

Cardiomyocyte mitophagy was detected by Mitophagy Detection Kit (Dojindo). Cardiomyocytes were cultured in 37 °C with 100 nmol/L Mtphagy dyes for 30 min. Cells were wished 2 times with PBS, and then treated with 10 µmol/L CCCP for 8 h prior to induce mitophagy. Cells were suspended into single cells and subsequently subjected to FCM to determine the flourecence intensity at 700 nm.

### Mitochondrial morphology

The mitochondria of cardiomyocytes were visualized using adenovirus carrying Cox8a-RFP,which has sequences of mitochondrial matrix localization derived from Cox8a. The differentiated 30-day CMs were digested into single cells and were inoculated in a laser confocal dish at a suitable density for four days with DMEM + 10% FBS. The CMs were then cultured in DMEM + 10% FBS containing Cox8a-RFP for 24 h. After 24 h, the original liquid was discarded and replaced with DMEM + 10% FBS. The mitochondria of cardiomyocytes were photographed within 24 h.

### Seahorse bioenergetics analyses

The mitochondrial respiratory capacity of cardiomyocytes was determined by Seahorse XFe96 Analyzer (Agilent Technologies). The differentiated 34-day CMs were plated in 96-well plates at 2 × 10^4^ cells/well at 37 °C for 24 h. The cells were incubated in DMEM containing 0.584 g/L L-glutamine and 5.5 mmol/L D-glucose for 1 h at 37 °C before analysis. 0.5 mg/mL oligomycin, 1 mmol/L FCCP, 1 mmol/L rotenone and 2 mmol/L antimycin A were sequentially added into each assigned well at indicated time point.

### Calcium transient

The calcium flux was determined by laser confocal Ca^2+^ imaging technique to evaluate intracellular calcium transient. The autonomous beating cardiomyocytes labeled by 5 μmol/L Cal-520AM and 0.02% Pluronic F-127 (AAT Bioquest) were detected by LSM-710 laser scanning confocal microscope (Carl Zeiss) with a detection time of 10 milliseconds under the line scanning mode in Zen software. Zen software was used to record calcium transient and MATLAB software (MathWorks) was performed to analysis calcium imaging signals.

### Microelectrode array (MEAs) analysis

The cardiomyocytes were digested at 32 days after induction and then inoculated to the surface of the coated MEAs probe. The myocardial cell field potential waveforms were recorded using a MEA2100 system at 35 days post induction. A bright-field signal video was recorded using a heated MC-rack to ensure that the cells were maintained at 37 °C. The data of field potential waveforms were quantitatively analyzed by using the Spike2 7.19 software (CED, UK).

### Contraction force measurements

2–3 days before the measurement, the cardiomyocytes were digested and planted on a confocal dish coated with Matrigel. The contractility of cardiomyocytes was detected by using video-based motion edge detection system. Through a Zeiss CFM-500 inversion fluorescence microscope and Video Sarcomere Length software (900B: VSL, Aurora Scientific), beating cardiomyocytes were clearly projected onto the screen. The spontaneous contraction traces of cardiomyocytes were recorded by using data analysis software (FelixGX, PTI). During the testing process, these cells were kept in 37 °C using heating equipment, and only the cardiomyocytes of the digestive single were analyzed.

### Plasmid construction

The pGL3 Basic plasmid was digested with two restriction endonucleases Hind III and Nhe I. Subsequently, the 2,000 bp fragment, the 363 bp fragment and the mutated 362 bp fragment of the 5′ upstream sequence of the Bnip3 gene were cloned into the pGL3 basic vector (Promega, Madison, WI). The open reading frame (ORF) of the Clock gene was cloned to pcDNA 3.1 by NotI and Apa 1 endonuclease. The ORF of the *BMAL1* gene was cloned to pCMV using BamHI and EcoRI. The plasmid constructed above was added to the DH5α to be transformed and cultured on ampicillin agar plate. The positive clones were selected for amplification, and the plasmid was extracted by using MN large pumping kit, and the concentration was measured. The existence of the *CLOCK*, *BMAL1* and *BNIP3* genes were confirmed by sequencing following plasmid extraction.

### Dual luciferase reporter assay

The following plasmids were instantly transfected into the HEK293T cells in the 6-well plates by means of LipofectamineTM 2000 regent (Invitrogen,USA) according different distribution combinations. These plasmids included 500 ng of pGL3-*BNIP3* plasmid, 100 ng of pRL-TK plasmid, 500 ng pcDNA 3.1, 500 ng pCMV, 500 ng *BMAL1* plasmid, and 500 ng *CLOCK* plasmid. The cells were harvested after 24 h of transfection and the activity of firefly and renilla luciferase was detected by the Dual Luciferase Reporter Gene Assay Kit (Beyotime, China). The firefly luciferase activity was corrected by the renilla luciferase activity.

### ChIP

ChIP was performed as described previously (Lan et al., 2007). The whole cell extracts were sonicated and immunoprecipitated with antibody of BMAL1 (CST). Real-time quantitative PCR was used to analyze the precipitated DNA samples.

### Statistical analysis

For statistical analysis, all data were expressed using mean ± SD. Student’s *t* test was used for comparison between the two groups. The statistical significance of differences between the experimental groups and several timepoints was determined using 2-way ANOVA with post-hoc test. *P*-values less than 0.05 was considered to be statistically different. All experimental results were analyzed using SPASS 17.0. GraphPad Prism 5 and Adobe illustrator software were used for analysis and graphing.

## Electronic supplementary material

Below is the link to the electronic supplementary material.

Supplementary material 1 (PDF 1619 kb)

Supplementary material 2 (MP4 2997 kb)

Supplementary material 3 (MP4 1999 kb)

Supplementary material 4 (MP4 495 kb)

Supplementary material 5 (MP4 581 kb)
