# Supplementary material for: BMAL1 regulates mitochondrial fission and mitophagy through mitochondrial protein BNIP3 and is critical in the development of dilated cardiomyopathy
Source: Protein Cell. 2020 Apr 10;11(9):661–79. doi: 10.1007/s13238-020-00713-x (PMC7452999; doi:10.1007/s13238-020-00713-x)
Supplement: Supplementary file 1 — Supplementary material 1 (PDF 1619 kb) [file 13238_2020_713_MOESM1_ESM.pdf]

SUPPLEMENTARY MATERIALS

Echocardiography

Use hair removal cream to obtain a clearer echo image to remove the chest hair of mice. The Mice were anesthetized with isoflurane gas and pushed into the preheating echo transmission platform. At the papillary muscle level, M-mode images were obtained from the parasternal long-axis view using a Vevo 2100 High-frequency imaging platform (VisualSonics Inc). Two experienced ultrasound physicians conducted a single blind test of cardiac ultrasound in mice. Left ventricular internal dimension at end systole (LVIDs), left ventricular internal dimension at end diastole (LVIDd), left ventricular posterior wall thickness at end systole (LVPWs), left ventricular posterior wall thickness at end diastole (LVPWd), interventricular septal thickness at end systole (IVSs), interventricular septal thickness at end diastole (IVSd), left ventricular mass were measured from M-mode tracings, and the average of three consecutive cardiac cycles is reported. The left ventricular fractional shortening percentage was calculated as ([LVIDd-LVIDs]/LVIDd)×100%. The left ventricular ejection fraction percentage was calculated as $([(LVIDd)^3-(LVIDs)^3]/(LVIDd)^3) \times 100\%$.

Quantitative real-time PCR

Cells were stored in RNA stabilization reagent (Invitrogen) at -80°C. Total RNA was extracted using the TRIzol plus RNA purification kit (Invitrogen). ReverTra Ace qPCR RT Master Mix (Toyobo) was an efficient and convenient kit for the synthesis of short-chain cDNA, carried out with 1 mg of total RNA. Real-time PCR was done using the Hieff qPCR SYBR Green Master Mix (YEASEN) based on the manufacturers' protocol on a Light-Cycler 96 System (Roche). The expression level of each gene was analyzed by △△Ct method, and all the data were normalized to GAPDH. Primer sequences can be found in table 5.

Western Blot

To obtain the total protein, RIPA buffer (50 mM Tris/pH 8.0, 150 mM NaCl, 2 mM EGTA, 1 mM EDTA, 1% NP-40, 0.5% sodium deoxycholate, 0.1% SDS) was used to obtain the whole cell lysate. Proteins were quantified by BCA Protein Assay Kit (TIANGEN, China). The equal amount of protein was run in Tris-glycine SDS-PAGE gels and then transferred to polyvinylidene fluoride (PVDF) membrane (Millipore, USA). At room temperature, membranes were blocked for 1 h in 5% skim milk in Tris-buffered saline (20 mM Tris/pH 7.6,150 mM NaCl, 0.1% Tween-20) and then incubated in primary antibody diluted in 5% skim milk overnight at 4°C. Primary antibodies used were found in table 4. Membranes were gently washed three times with PBS-T buffer for 10 minutes at a time, then incubated with horseradish peroxidase-conjugated secondary antibodies for 1 hour at room temperature. Membranes were washed again in PBS-T, the method is the same as above, then developed with Clarity Western ECL Substrate (Bio-Rad Laboratories, #170–5061) and imaged using an imaging system (Tanon, China). Densitometry was analyzed using ImageJ software (National Institutes of Health).

Transmission electron microscopy

The CMs were removed from the petri dish using a cell scraper, rinsed with PBS, pelleted at 800 rpm for 20 mins, and then fixed in 2.5% glutaraldehyde in PBS for 2 hours at 4°C. Pellets were wished 3 times with PBS, and then post-fixed with 1% osmium tetroxide in PBS for 2 hours on the rotator. The pellets were wished again before dehydration in a range of increasing ethanol concentrations (30%, 50%, 70%, 85%, 95%, 100%, 100%, 100%). After dehydration, pellets were infiltrated in a series of acetone with increasing concentrations (33%, 66%, 100%, 100%) and then polymerized in 60°C for 24 hours. The resin blocks were cut into 50-60 nm thick resin

block, partially mounted on 300 mesh copper grids (Electron Microscopy Sciences). The slices were stained positive with uranyl acetate and lead citrate and were observed by a PHILIPS CM-120 transmission electron microscope (PHILIPS).

Immunofluorescence and Alkaline phosphatase staining

The cells were rinsed with PBS and fixed with 4% paraformaldehyde for 5 minutes. The cells were permeabilized with 0.05% Triton X-100 at room temperature for 15 minutes, and washed three times with PBS, and then incubated in 4% goat serum for 30 minutes. The cells were stained by primary antibodies overnight at 4°C.The next day, the cells were incubated with Alexa Fluor conjugated secondary antibodies at 37°C for 1 hour, and then counterstained with DAPI at room temperature for 5 minutes. Differentiated cardiomyocytes were recognized by immunostaining of cardiac troponin T (cTnT, Thermo Scientific, Table 3). Pluripotency of Bmal1 gene deficient hESC line 7 was validated by immunostaining of SOX2 (Abcam), OCT4 (Santa cruz), SSEA4 (Abcam) and NANOG (Abcam). The antibody information could be found in Table 3. According to the manufacturer's instruction, Alkaline phosphatase Kit (Millipore) was used to identify the pluripotency. The stained cells were imaged by a Leica DMi8 microscope. The size of cell surface area was quantified with ImageJ software package (National Institutes of Health).

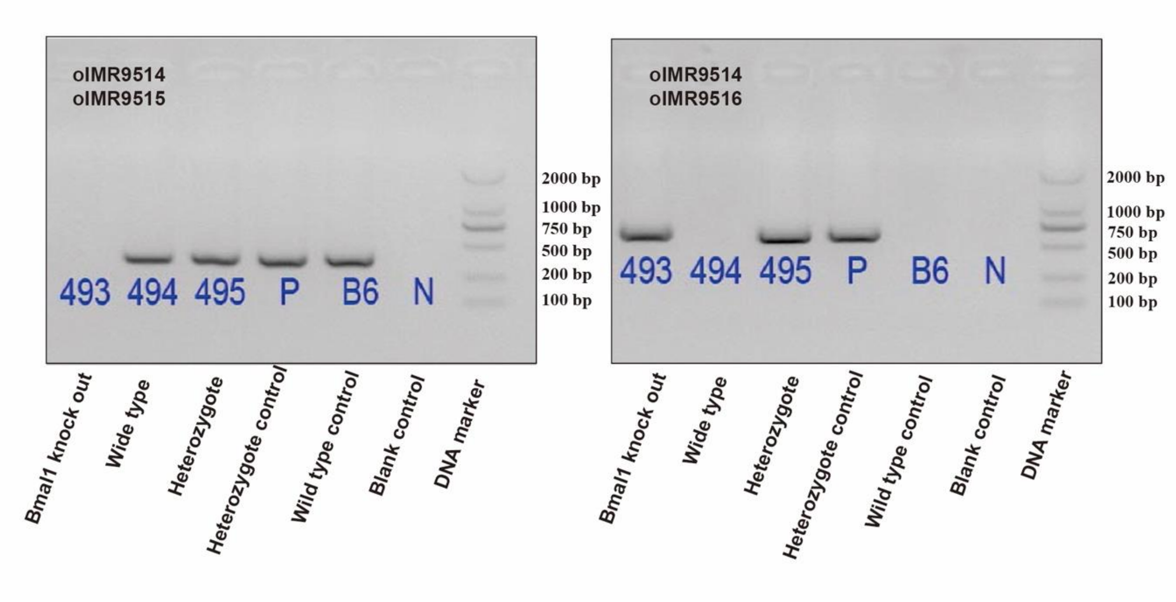

Supplementary Figure 1. Genotyping of *Bmal1* knockout mouse by agarose gel electrophoresis. N is a blank control group. B6 is a known wild-type control mouse(C57BL/6J), and P is a known heterozygous control mouse. The electrophoresis band located at 600 bp (see right-hand patterning) is for the *Bmal1* gene knockout mice (*Bmal1* knock Out, No. 493), 399 bp (see left-hand patterning) is for wild mouse (Wild type, No. 494). Both 399 bp (see left-hand patterning) and 600 bp (see right-hand patterning) are for hybrid mice (Heterozygote, No. 495).

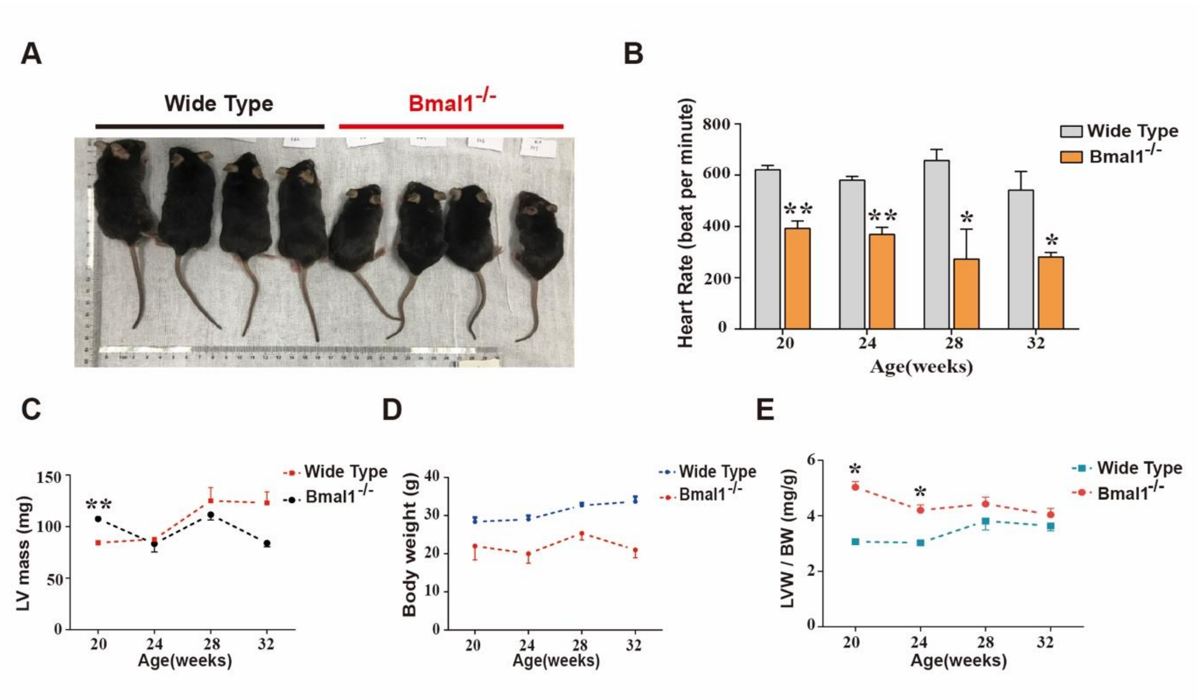

Supplementary Figure 2. *Bmal1*-deficient mice showed decreased body weight and LV mass. (A) *Bmal1* knockout mice were shorter in length comparing with age- and sex-matched wild type group. (B) *Bmal1* KO mice were lighter in weight in groups at any age. (C) LV mass was evaluated by echocardiogram. Wild type mice showed a gradual increase in LV mass by age, while *Bmal1* KO mice exhibited a decrease in LV mass from 20 weeks to 32 weeks after birth. (D) *Bmal1* KO mice exhibited significantly greater LV mass/body weight ratio at the age of 20 weeks and 24 weeks. Data were represented as Mean ± SD. n = 4. *$p < 0.05$ and **$p < 0.01$ versus control by two-tailed Student's t test.

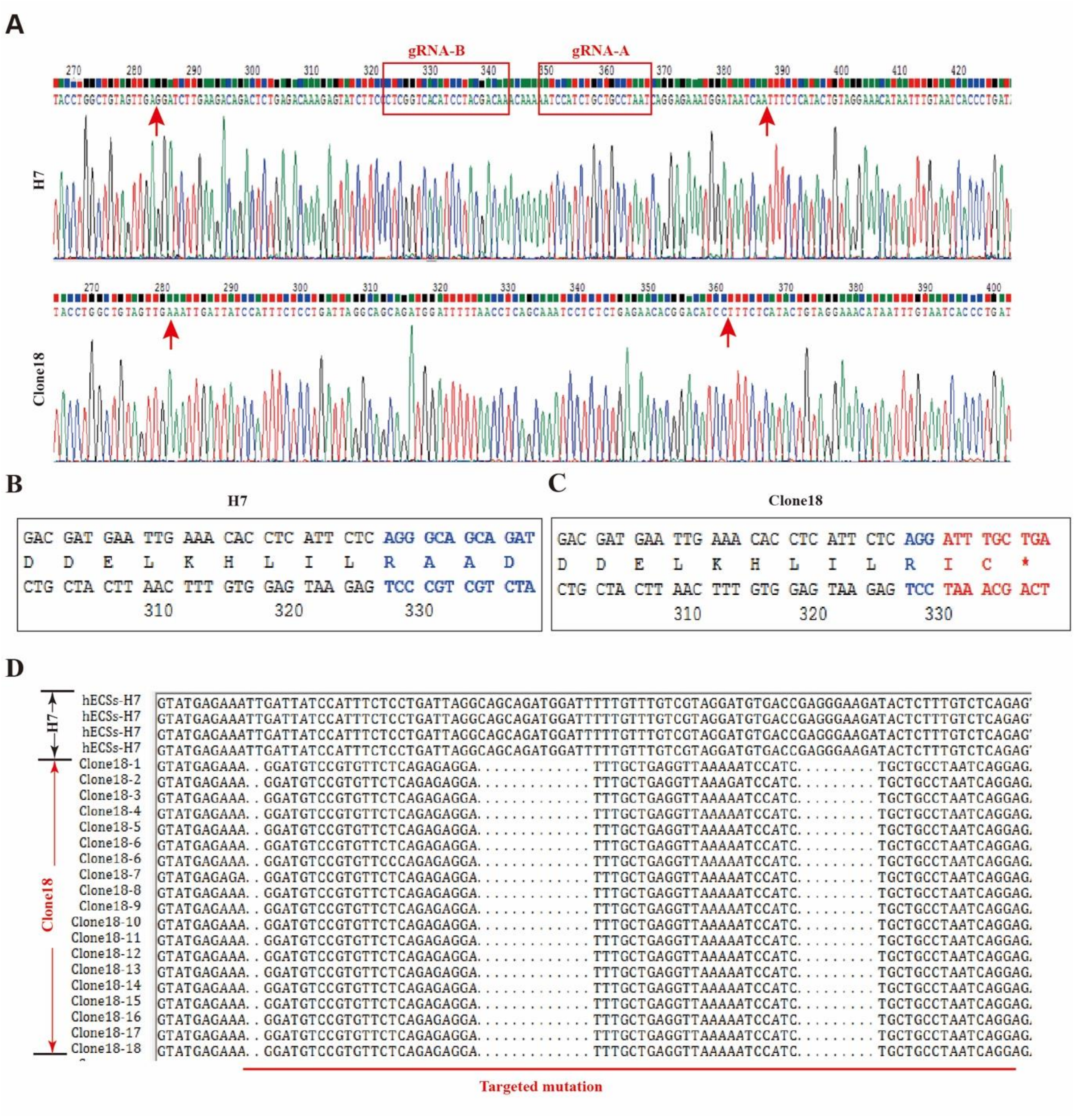

Supplementary Figure 3. Establishment of the *BMAL1* deficient H7-hESCs. (A) Sanger's sequencing of PCR-amplified genomic DNA in wild type and *BMAL1* KO hESCs. (B) Indel mutations occurred within the targeted region of *BMAL1* led to frameshifts and creation of a premature stop codon (stop-gained) in sequenced clones. (C) The amplified PCR product of target cutting region was directly cloned into a linearized T-vectors. Both alleles had the same genetic modification.

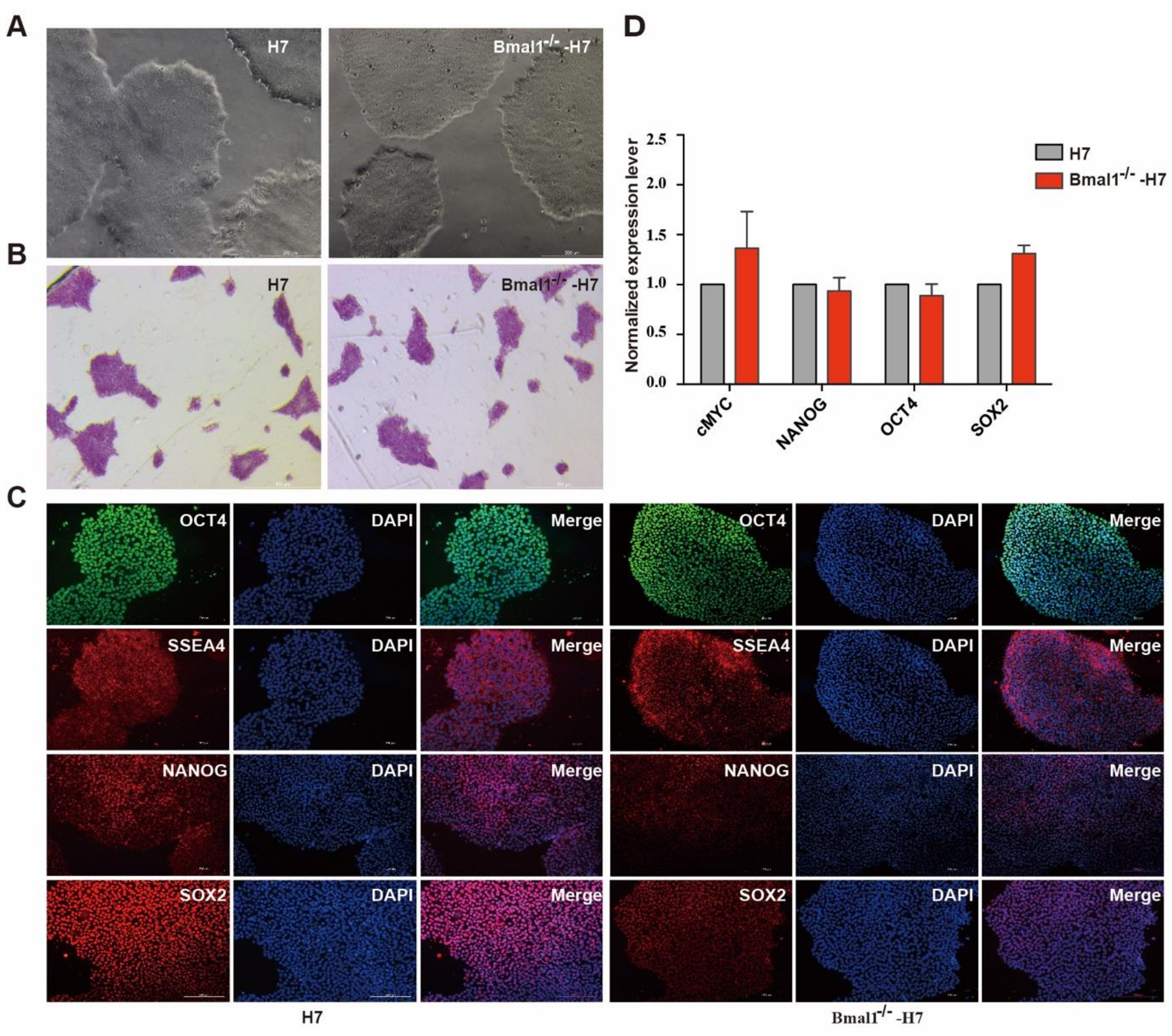

Supplementary Figure 4. Verification of pluripotency for the _BMAL1_ KO hESCs. (A) The colony morphology of _BMAL1_ KO H7 kept the characteristic of normal human embryonic stem cells. Using genetic reprogramming with CRISPR/Cas9, ESC-like pluripotent capabilities had not been disturbed. (B) Alkaline phosphatase staining of wildtype and _BMAL1_ KO H7 hESCs. (C) Immunofluorescence staining of pluripotency markers OCT4，SSEA4，NANOG and SOX2 in wildtype and _BMAL1_ KO H7 hESCs. (D) qPCR of pluripotent gene expression in wildtype and _BMAL1_ KO H7 hESCs. Data were represented as Mean ± SD.

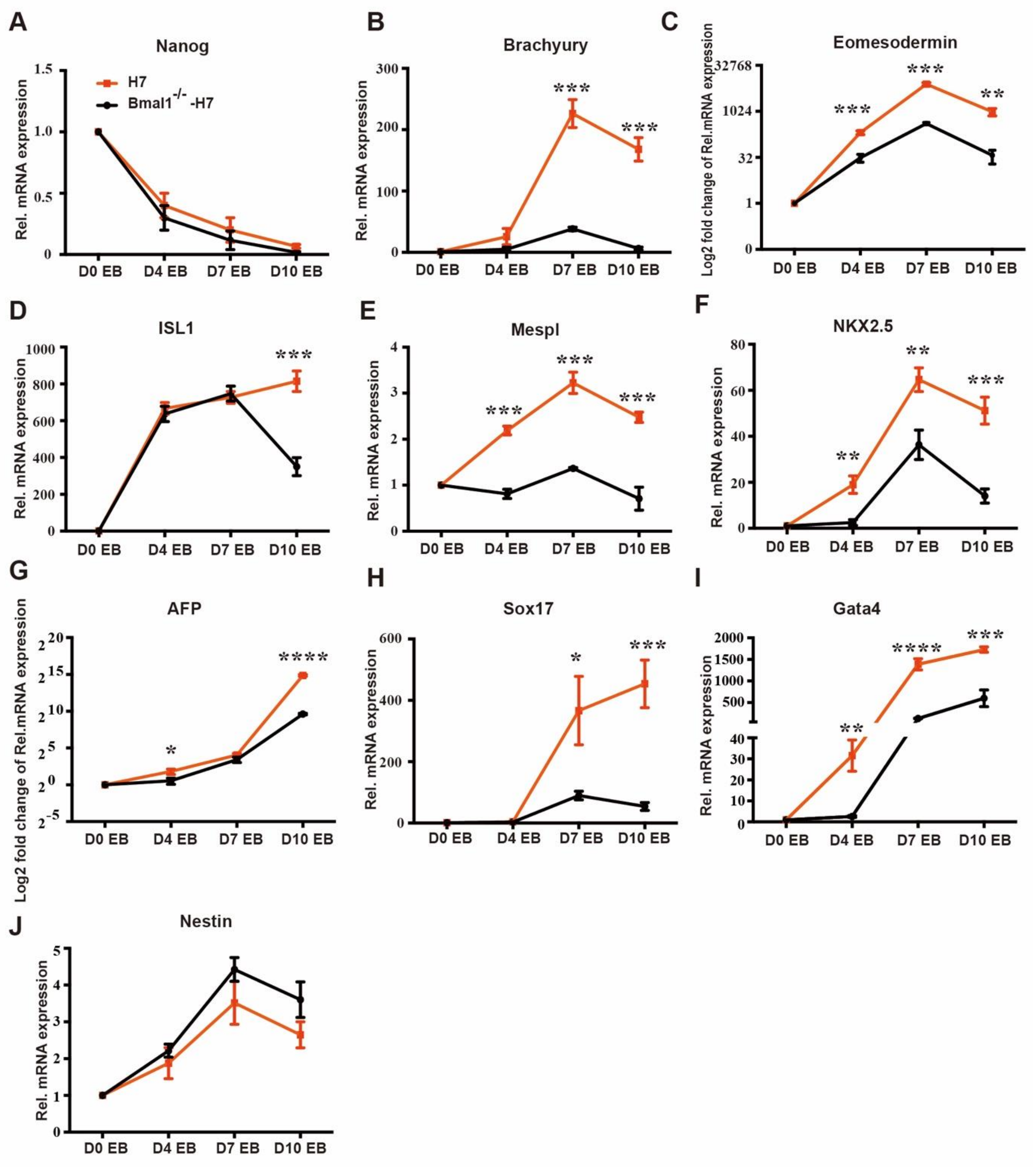

Supplementary Figure 5. QPCR analyses of different marker genes from the three embryonic germ layers in spontaneous differentiation of wildtype and *BMAL1* KO H7 hESCs. Data were Mean ± SD, n = 3, * $p < 0.05$, ** $p < 0.01$, *** $p < 0.001$, **** $p < 0.0001$. Two-tailed Student's t test.

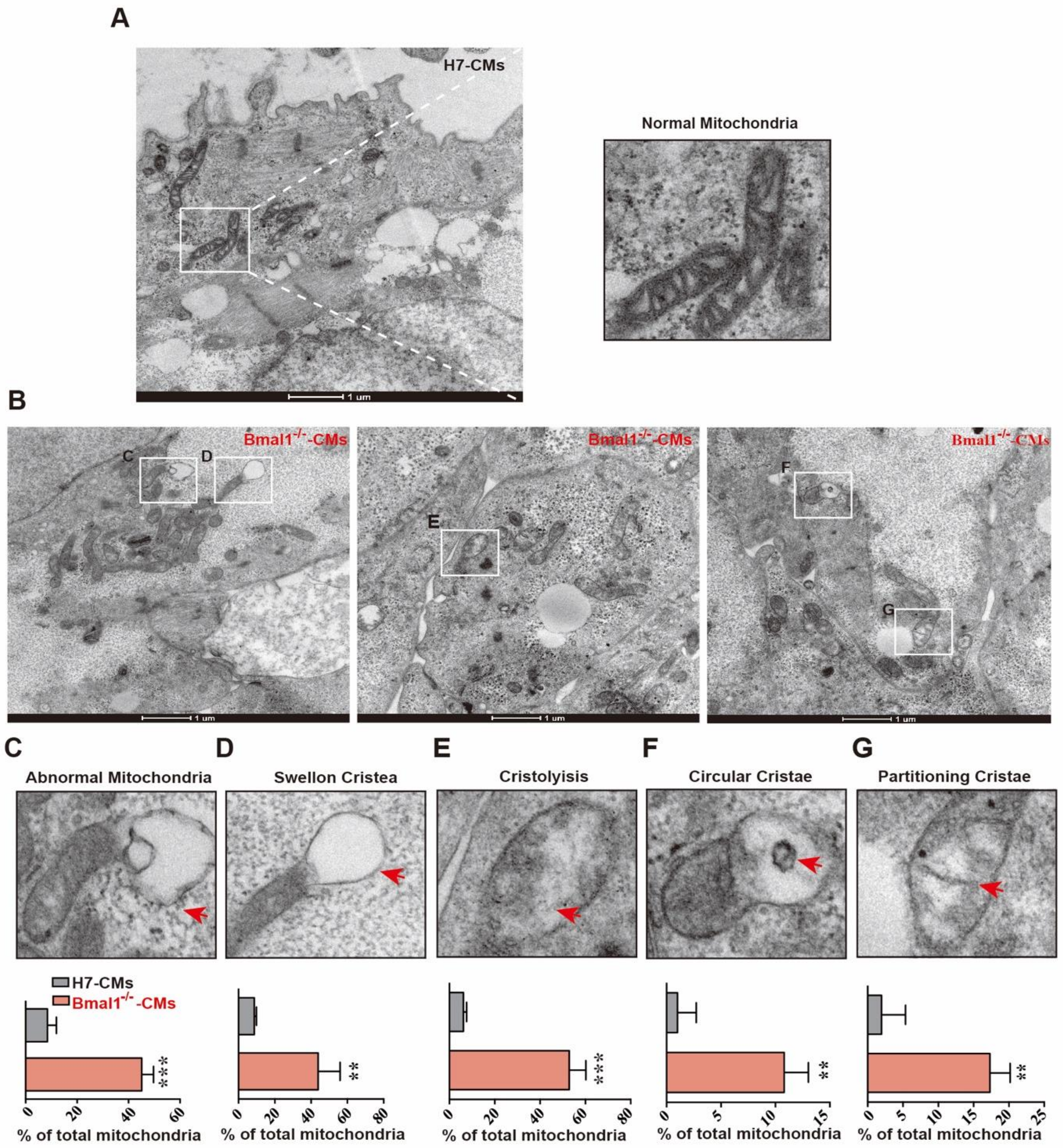

Supplemental Figure 6. Morphology of mitochondrial cristaes in wildtype and _BAML1**_ KO hESC-derived cardiomyocytes.** (A) Wildtype hESC-derived cardiomyocytes showed normal mitochondrial cristaes. (B) Representative mitochondrial cristae abnormalities in _BMAL1_ KO hESC-derived cardiomyocytes. (C-G) Higher magnification of the white boxes in (B) showing different types of aberrant

mitochondrial cristaes (Red arrows) and its percentage relative to total mitochondira. Data represented Mean ± SD. ** $p < 0.01$ and **** $p < 0.0001$ versus H7-CMs by two-tailed Student's t test.

Supplementary Movie 1. Spontaneous beating of wildtype hESC-derived cardiomyocytes day 35 post differentiation.

Supplementary Movie 2. Spontaneous beating of *Bmal1* KO hESC-derived cardiomyocytes day 35 post differentiation. *Bmal1* KO cardiomocytes showed a reduced and irregular beating frequency.

Supplementary Movie 3. Mitochondria of wildtype hESC-derived cardiomyocytes day 35 post differentiation.

Supplementary Movie 4. *Bmal1* KO hESC-derived cardiomyocytes exhibited diminished mitochondrial fission comparing with that in wildtype hESC-cardiomyocytes.

TABLES

Table 1. Analyses of E-Box structure in the promoter region of human *Bnip3* gene

***Bnip3* gene Promoter**	**Classic E-Box**
39-44 bp	CACGTG
934-939 bp	CACTTG
1326-1331 bp	CAGGTG
1536-1541 bp	CAAATG
1563-1568 bp	CAAGTG
1742-1747 bp	CATTTG
1793-1798 bp	CATATG
	**Non-classical E-Box**
1033-1038 bp	CAGCTT

Table 2. Antibodies used in flow cytometry

**Antibody**	**Source**	**Vendor**	**Cat. No.**
Cardiac troponin T	Mouse	Thermo Scientific	MA5-12960
Secondary antibody	PE goat anti mouse	Biolegend	405307

Table 3. Antibodies used in immunofluorescence staining

**Antibody**	**Source**	**Vendor**	**Cat. No.**
Sarcomeric α-actinin	Mouse	Abcam	18061
Cardiac troponin T	Rabbit	Abcam	45932
NANOG	Mouse	Santa Cruz	293121
SSEA4	Mouse	Abcam	16287
OCT4	Rabbit	Santa Cruz	9081
SOX2	Mouse	Abcam	171380
Nuclei (DAPI)	-	Sigma Aldrich	D9542
Secondary antibody	Alexa Fluor 594 goat anti mouse	Life Technologies	A11005
Secondary antibody	Alexa Fluor 594 goat anti rabbit	Life Technologies	A11012
Secondary antibody	Alexa Fluor 488 goat anti mouse	Life Technologies	A11001
Secondary antibody	Alexa Fluor 488 goat anti rabbit	Life Technologies	A11008

Table 4. Antibodies used in western blot

Antibody	Source	Vendor	Cat. No.
BMAL1	Rabbit	CST	14020
LC3 A/B	Rabbit	CST	12741T
SQSTM1	Mouse	Abcam	56416
MFN2	Rabbit	Abcam	50838
BNIP3	Rabbit	Abcam	109362
β-actin	Mouse	Proteintech	60008-1-Ig
Secondary antibody	Goat Anti Rabbit IgG(H+L)	Proteintech	00001-2
Secondary antibody	HRP-Goat Anti-Mouse IgG (H+L)	Proteintech	00001-1

Table5 Primer sequences for Quantitative real-time PCR

Gene Name	NCBI Gene ID	Primer (5'-3')
GAPDH	2597	Forward: GGAGCGAGATCCCTCCAAAAT<br>Reverse:GGCTGTTGTCATACTTCTCATG G
Brachyury	778911	Forward: AAGGAGCTCACCAATGAGATG<br>Reverse: TGACTTTGCTGAAGGAGACG
Eomesodermin	8320	Forward: AATGTGTTCGTAGAGGTGGTG<br>Reverse: AGCGGTGTACATGGAATCATAG
ISL1	3670	Forward: CTGTGCTGAACGAGAAGCAGC<br>Reverse: GTAAGCCACCGTCGTGTCTCTC
Mesp1	55897	Forward: CTGCCTGAGGAGCCCAAGTGA<br>Reverse: TCACTTGGGCTCCTCAGGCAG
NKX2.5	1482	Forward: GCCGCCAACAACAACTTC<br>Reverse: TACCAGGCTCGGATACCAT
AFP	174	Forward: GAGATGTGCTGGATTGTCTGC<br>Reverse: GCAAGCTGAGGATGTCTTCTTG
GATA4	14463	Forward: ACCAC AAGATGAACGGCAT<br>Reverse: CGTGGAGCTTCATGTAGAGG
SOX17	64321	Forward: TGAACGCTTTCATGGTGTGG<br>Reverse: TGTAGTTGGGGTGGTCCTGC
Nestin	10763	Forward: CTGCGGGCTACTGAAAAGTTCC<br>Reverse: GCTGGCACAGGTGTCTCAAG
c-MyC	4609	Forward: TACAACACCCGAGCAAGGAC<br>Reverse: GAGGCTGCTGGTTTTCCACT
Nanog	79923	Forward: CTTCACCTATGCCTGTGATTTG<br>Reverse: GCTGAGGTATTTCTGTCTCTG

Oct4	5460	Forward: GCAAAGCAGAAACCCTCGTG<br>Reverse: CACACTCGGACCACATCCTT
Sox2	6657	Forward: ATGGACAGTTACGCGCACAT<br>Reverse: CGAGCTGGTCATGGAGTTGT
